# Combining a CD20 Chimeric Antigen Receptor and an Inducible Caspase 9 Suicide Switch to Improve the Efficacy and Safety of T Cell Adoptive Immunotherapy for Lymphoma

**DOI:** 10.1371/journal.pone.0082742

**Published:** 2013-12-17

**Authors:** Lihua E. Budde, Carolina Berger, Yukang Lin, Jinjuan Wang, Xubin Lin, Shani E. Frayo, Shaunda A. Brouns, David M. Spencer, Brian G. Till, Michael C. Jensen, Stanley R. Riddell, Oliver W. Press

**Affiliations:** 1 Department of Hematology and Hematopoietic Cell Transplantation, City of Hope, Duarte, California, United States of America; 2 Clinical Research Division, Fred Hutchinson Cancer Research Center, Seattle, Washington, United States of America; 3 Departments of Medicine and Bioengineering, University of Washington, Seattle, Washington, United States of America; 4 Pathology and Immunology, Baylor College of Medicine, Houston, Texas, United States of America; 5 Bellicum Pharmaceuticals, Inc., Houston, Texas, United States of America; 6 Center for Immunity and Immunotherapies, Seattle Children's Research Institute, Seattle, Washington, United States of America; 7 Institute for Advanced Study, Technical University of Munich, Munich, Germany; Saint Louis University School of Medicine, United States of America

## Abstract

Modification of T cells with chimeric antigen receptors (CAR) has emerged as a promising treatment modality for human malignancies. Integration of co-stimulatory domains into CARs can augment the activation and function of genetically targeted T cells against tumors. However, the potential for insertional mutagenesis and toxicities due to the infused cells have made development of safe methods for removing transferred cells an important consideration. We have genetically modified human T cells with a lentiviral vector to express a CD20-CAR containing both CD28 and CD137 co-stimulatory domains, a “suicide gene” relying on inducible activation of caspase 9 (iC9), and a truncated CD19 selectable marker. Rapid expansion (2000 fold) of the transduced T cells was achieved in 28 days after stimulation with artificial antigen presenting cells. Transduced T cells exhibited effective CD20-specific cytotoxic activity *in vitro* and in a mouse xenograft tumor model. Activation of the iC9 suicide switch resulted in efficient removal of transduced T cells both in vitro and in vivo. Our work demonstrates the feasibility and promise of this approach for treating CD20^+^ malignancies in a safe and more efficient manner. A phase I clinical trial using this approach in patients with relapsed indolent B-NHL is planned.

## Introduction

Non-Hodgkin’s lymphoma (NHL) is the seventh most common cause of cancer in the United States in 2012 [1]. Conventional therapies including radiotherapy, combination chemotherapy, and biologics do not cure most patients with NHL. The CD20 antigen is a B-cell--specific surface molecule whose expression spans the pre-B to mature B-cell stages and is minimally internalized or shed. More than 95% of B-NHL cells express CD20 and the majority of relapsed B-NHL cells retain surface CD20 expression despite repetitive anti-CD20 antibody exposure. Antigen escape is not a major mechanism for resistance to rituximab, a commonly utilized anti-CD20 antibody used to treat CD20^+^ NHLs. All these features make CD20 an excellent tumor-associated target for T cell-based therapy.

Adoptive cellular therapy using T cells genetically modified to express a chimeric antigen receptor (CAR) against a tumor-associated antigen is an emerging immunotherapeutic approach for a variety of malignancies including lymphoma and leukemia [2]–[5]. The CAR molecule when expressed on a T cell possesses two essential properties. First, it redirects the specificity of T cells in an MHC-independent fashion via an N-terminal single chain variable fragment (scFv) specific for a tumor-associated surface antigen. Second, it transmits an activation signal via a C-terminal endodomain, typically the CD3ζ chain of the T-cell receptor complex. Preclinical studies and clinical trials have demonstrated that therapy with CAR-modified T cells lacking co-stimulatory signals is safe and feasible, but also revealed suboptimal activation, expansion and persistence of the T cells [6]–[9]. “Second generation” CARs that contain a co-stimulatory domain derived from CD28, CD137 (4-1BB), or OX40 placed in cis with the CD3ζ endodomain may exhibit improved antigen-specific cytokine production, proliferation, cytotoxicity, and persistence [10]–[14]. We and others have shown that incorporation of two co-stimulatory domains can further potentiate the function of CAR-modified T cells in preclinical studies [15]–[17]. We have recently conducted a pilot clinical trial using CD20-CAR T cells that contained two co-stimulatory domains from CD28 and CD137 (4-1BB), in 4 patients with indolent NHL or mantle cell lymphoma[18]. Our study demonstrated feasibility and the T cells modified with this third generation CD20-CAR had improved persistence compared to a previous trial using T cells modified with a CD20-CAR that lacked co-stimulatory domains (12 months versus 2 - 3 weeks). Despite these promising findings, several problems hindered the further exploitation of this plasmid-based gene transfer approach, including low levels of CAR expression, the laborious process of generating and expanding CD20-CAR T cells, and limited efficacy. Therefore better gene-transfer technologies and more efficient cell production methods are needed to fully exploit third generation CD20-CAR T cells.

One potentially devastating risk of gene therapy is insertional mutagenesis. This complication has caused at least 3 deaths in hematopoietic stem cell gene therapy trials for severe combined immunodeficiency [19], [20]. In addition, persistent B cell aplasia, and cytokine storm are common in clinical trials using CD19-CAR T cells [21] and one death from multi-organ failure was observed with ERBB2-CAR T cells [22]. These serious adverse events have led to wider recognition of the importance of incorporating an inducible suicide gene in the transferred cells. One such gene, designated iC9, encodes a fusion protein that links a truncated human caspase 9 lacking the endogenous caspase recruitment domain (CARD) with a mutated FK506-binding protein (FKBP12) mediating dimerization. In the presence of a biologically inert prodrug, AP1903, functional active caspase 9 is generated, leading to initiation of the mitochondrial apoptotic cascade, and ultimately death of the transferred cells [23]. The iC9 system efficiently eliminated T cells modified to express a CD19-CAR containing a CD28 co-stimulatory domain, and IL-15 [24]. Di Stasi et al. also demonstrated the ability of the iC9 system to effectively remove transferred T cells causing graft-versus-host disease in a phase I clinical trial [25].

Here we describe the engineering of genetically modified T cells using a highly efficient lentiviral-mediated gene transfer approach to express a third generation CD20-CAR containing both CD28 and 4-1BB co-stimulatory domains, an inducible iC9 and a truncated CD19 (Δ19) selectable marker.

## Materials and Methods

### Ethics Statement

Human peripheral blood mononuclear cells (PBMC) were obtained by apheresis from healthy donors who provided written informed consent according to a cell procurement protocol approved by the Fred Hutchinson Cancer Research Center Institutional Review Board (protocol 868). Murine experiments were conducted according to a protocol that was pre-approved by the Institutional Animal Care and Use Committee (IACUC) at the Fred Hutchinson Cancer Research Center (protocol number: 1490). These studies were performed in strict accordance with the recommendations in the Guide for the Care and Use of Laboratory Animals of the National Institutes of Health and all efforts were made to minimize suffering.

### Lentiviral vector construction and lentiviral production

**Figure 1:**
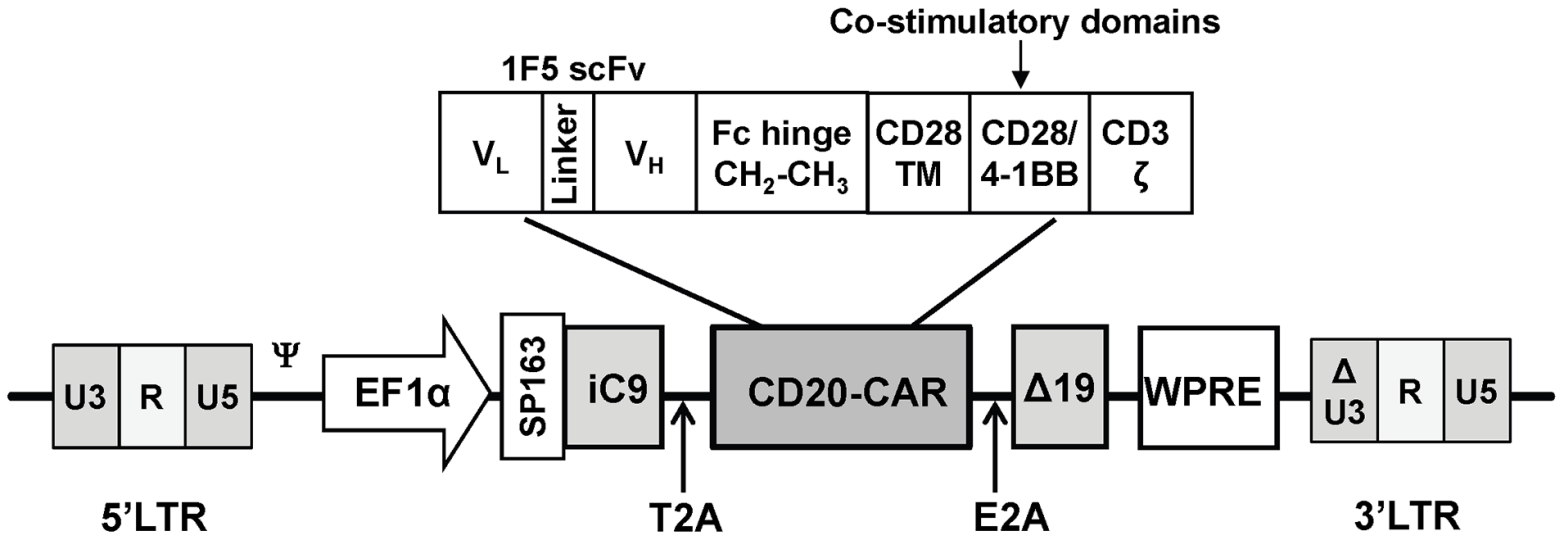
Schematic diagram of the iC9-CD20CAR-Δ19 lentiviral vector. The CD20-CAR encompasses a scFv derived from the 1F5 antibody linked to a human IgG1 Fc (hinge region, CH2 and CH3), a CD28 transmembrane domain (CD28 TM), a CD28 co-stimulatory domain, a 4-1BB co-stimulatory domain, and a CD3ζ intracellular domain. SP163, a translational control element; iC9, inducible caspase 9-based suicide gene; Δ19, a truncated CD19 shortening of the CD19 intracellular domain to only 19 amino acids and removal of all tyrosine residues; T2A, *Thoseaasigna* virus 2A peptide sequence; E2A, equine rhinitis A virus 2A peptide sequence.

We utilized a lentiviral vector (SINpWPT-GFP provided by Dr. Hans-Peter Kiem, Fred Hutchinson Cancer Research Center (FHCRC, Seattle, WA)) containing an internal elongation factor 1α (EF-1α) promoter and a woodchuck posttranscriptional regulatory element (WPRE) [26]. The CD20-CAR gene consisted of a Kozak consensus ribosome-binding sequence, a murine κ chain signal peptide, a sequence encoding the V_L_ region of the murine 1F5 antibody [27], an 18-residue linker peptide, a sequence encoding the V_H_ region of the 1F5 antibody, an IgG1 hinge-CH2-CH3 domain, the transmembrane region of the human CD28 molecule, an intracellular signaling domain containing both CD28 and 4-1BB domains, and the signaling domain of CD3ζ. A human Δ19 which shortens the CD19 intracellular domain to only 19 amino acids and lacks all conserved tyrosine residues that serve as phosphorylation sites [28] was employed as a selectable marker. The CD20-CAR gene, the iC9 gene (provided by Dr. David Spencer, GenBank NM001229), and the Δ19 gene were linked together using 2A sequence peptides [29] and cloned into the SINpWPT-GFP backbone to generate the iC9-CD20CAR-Δ19 lentiviral vector (Figure 1). Lentiviral vectors were pseudotyped with VSV-G envelope and produced by transient transfection of 293T cells [30].

### Gamma-retroviral vector construction and production

Human CD20 was cloned as previously described [31]. The cDNA sequences of human CD80, LFA-3, and ICAM-1 (Open Biosystems, Pittsburgh PA) were cloned into the pMSCVpuro vector (BD Biociences, San Jose, California). All constructs were verified by sequencing. The vector encoding firefly luciferase (FFLuc) used for in vivo imaging has been described previously [31]. The Phoenix G packaging cell line was obtained from Dr. Gary Nolan (Stanford University, http://www.stanford.edu/group/nolan/retroviral_systems/phx.html) and transiently transfected with pMSCVpuro vectors. Retroviral supernatants were obtained and concentrated using PEG.

### Generation of CAR T cells

Human peripheral blood mononuclear cells (PBMC) were negatively selected using immunomagnetic beads to obtain T cells (Miltenyi Biotec Inc., Auburn, CA). T cells were then activated using magnetic beads coated with anti-CD3 and anti-CD28 antibodies (Life Technologies, Grand Island, NY) at a 1∶1 ratio. Activated T cells were transduced daily for 2 days by spinoculation at 1200 g at 32°C for 1 hour in plates coated with retronectin (Clontech Laboratories, Inc., Mountain View, CA) using concentrated lentiviral supernatants supplemented with 8 µg/ml polybrene, and 50 U/ml of human rIL-2 (Chiron, Emeryville, California). Magnetic beads were removed between days 8 and 10. T cell expansion was achieved by co-culturing activated T cells with irradiated NIH3T3-based artificial antigen presenting cells (AAPCs) containing CD20 and costimulatory molecules. All AAPCs were irradiated (1500cGy) and plated 24 hours before use for T cell expansion. Freshly irradiated AAPCs were provided to T cells every 7 days.

### Cell lines

The Granta, Daudi, Raji, Ramos, Jurkat T cell and NIH3T3 lines were obtained from the American Type Culture Collection (ATCC, Manassas, VA). Raji-FFLuc tumor cells were generated by transducing Raji cells with a retroviral vector encoding the FFLuc gene [31]. A panel of artificial antigen-presenting cells (AAPCs) was constructed using the NIH3T3 cell line (ATCC) which was transduced with either a retroviral vector encoding human CD20 alone, or sequentially with two retroviral vectors encoding human CD20 and CD80, or four vectors encoding human CD20, CD80, ICAM-1, and LFA-3. NSO-IL15 cells were obtained from Dr. Michael Jensen (Seattle Children's Research Institute, Seattle, WA).

### Flow cytometric analysis

T cells were stained with a panel of antibodies including monoclonal mouse anti-human CD3, CD4, CD8, CD28, CD56, CD45RA, CD45RO, CD25 and CD62L antibodies (BD Biosciences, San Jose, California), a goat anti-mouse Fab-specific antibody (Sigma, Saint. Louis, MO), and a mouse anti-human IgG, Fc-specific F(ab’)^2^ antibody (Jackson Immuno Research, West Grove, PA). All samples were acquired with a BD FACSCanto (BD Biosciences, San Jose, California) and analyzed using FlowJo software (TreeStar, Ashland, OR).

### Western blotting

Whole cell lysates of T cells were generated, and subjected to western blotting as described [15].

### Cytotoxicity and cytokine secretion assays

The cytolytic activity of T cells was determined in a 4-hour chromium release assay as previously described [15]. In some experiments, a flow cytometric assay in which T cells were co-cultured with CD20^+^ Ramos tumor cells at E:T ratios of 2∶1 and 5∶1 was used to assess cytotoxicity. Residual tumor cells were distinguished from T cells using antibodies against CD22 (Ramos) and CD3 (T cells).T cells were co-cultured with target cell lines at an E:T ratio of 2∶1 for 48 hours. Production of cytokines in the supernatants was measured by enzyme-linked immunosorbent assays [18]. The in vitro cytotoxicity of AP1903-mediated T cell deletion was assessed by culturing T cells in the presence of various concentrations of AP1903 for 24 to 72 hours. The viable T cells were determined by trypan blue enumeration.

### Mouse xenograft studies

Six to ten-week-old NOD/SCID mice (Bernstein Laboratory, FHCRC, Seattle, WA) were injected intravenously (i.v.) with 5×10^5^ Raji-FFLuc cells via the tail vein. The tumor load was measured by in vivo imaging as previously described [31]. Non-transduced T cells or iC9-CD20CAR-Δ19 T cells (5×10^6^ each dose) were administered i.v. on days 2 and 9 following tumor injection as indicated. For the in vivo evaluation of the iC9 gene, NOD-SCID-γ_c_^−/−^ (NSG) mice (Charles River Laboratories International, Inc., Wilmington, MA) were injected intravenously (i.v.) with 5×10^5^ Raji-FFLuc cells via the tail vein on day 0. T cells transduced with iC9-CD20CAR-Δ19 (1×10^7^ each dose) were administered i.v. on days 2 and 7. Irradiated NSO-IL-15 cells (1.5×10^7^) were also administered i.p. every other day (QOD) to provide a source of human IL-15 in the mouse host. Two doses of AP20187 (Ariad Pharmaceuticals) were given i.p. on days 13 and 14. Control mice received two doses of PBS. Samples from blood, bone marrow, spleen and liver were collected 12 hours after the second injection and the presence of transferred T cells (CD19^+^CD22^−^) and tumor cells (CD19^+^CD22^+^) determined by flow cytometry [18].

### Statistical analysis

The statistical significance of differences between test samples was determined by using student’s *t* test.

## Results

### Efficient generation of iC9-CD20CAR-Δ19 T cells using lentiviral gene transfer

We generated T cells transduced with a self-inactivating (SIN) lentiviral vector (iC9-CD20CAR-Δ19) encoding iC9, CD20-CAR, and a Δ19 selectable marker (**Figure 1**). This vector was constructed using the EF-1α promoter to drive transgene expression and maintained high levels of CAR expression in transduced T cells.

**Figure 2:**
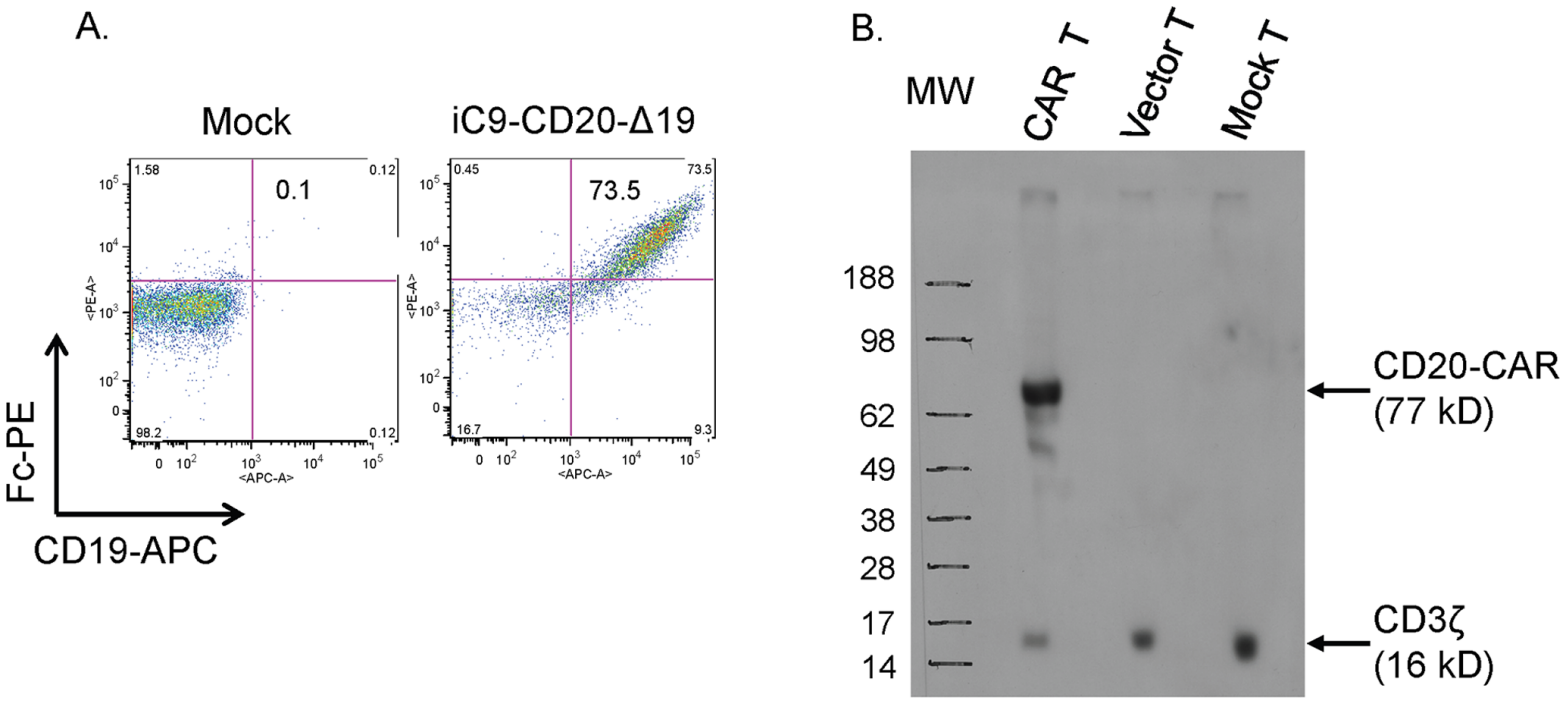
Expression of CD20-CAR. (**A**) Flow cytometric examination of surface expression of the CD20-CAR and CD19. Mock, non-transduced T cells. The CAR was detected with a PE-conjugated mouse anti-human IgG (Fc-specific) antibody. CD19 expression was detected with an APC conjugated mouse anti-human CD19 antibody. Cells were gated on total live cells 8 days after transduction. The insert value in the upper right quadrant indicates the percentage of CD20-CAR^+^ and CD19^+^ T cells. (**B**) Western blot analysis of CD20-CAR expression. Whole-cell lysates from transduced T cells (CAR T), T cells transduced with empty vector (vector T), and non-transduced T cells (mock T) were hybridized with mouse anti-human CD3ζ antibody under reducing conditions. The 16-kD band corresponds to the endogenous CD3ζ chain; the 77 kD band corresponds to the CD20-CAR.

The CD20-CAR contains an anti-CD20 scFv from the 1F5 antibody fused to human IgG1 hinge-CH2-CH3, a CD28 trans-membrane domain, CD28 and 4-1BB co-stimulatory domains, and a CD3ζ endodomain. Levels of CD20-CAR and ΔCD19 expression were detected by flow cytometric analysis in 10 to 75% of activated human T cells after lentiviral transduction (**Figure 2A**). The generation of CD20-CAR molecules was independently verified by western blotting using a mouse anti-human CD3ζ monoclonal antibody (**Figure 2B**). The expression level of the CD20-CAR exceeded that of the endogenous CD3ζ, demonstrating abundant CAR production by the lentiviral gene transfer method.

### Rapid expansion and enrichment of iC9-CD20CAR-Δ19 T cells using a NIH3T3-based artificial antigen presenting cell (AAPC) system

**Figure 3:**
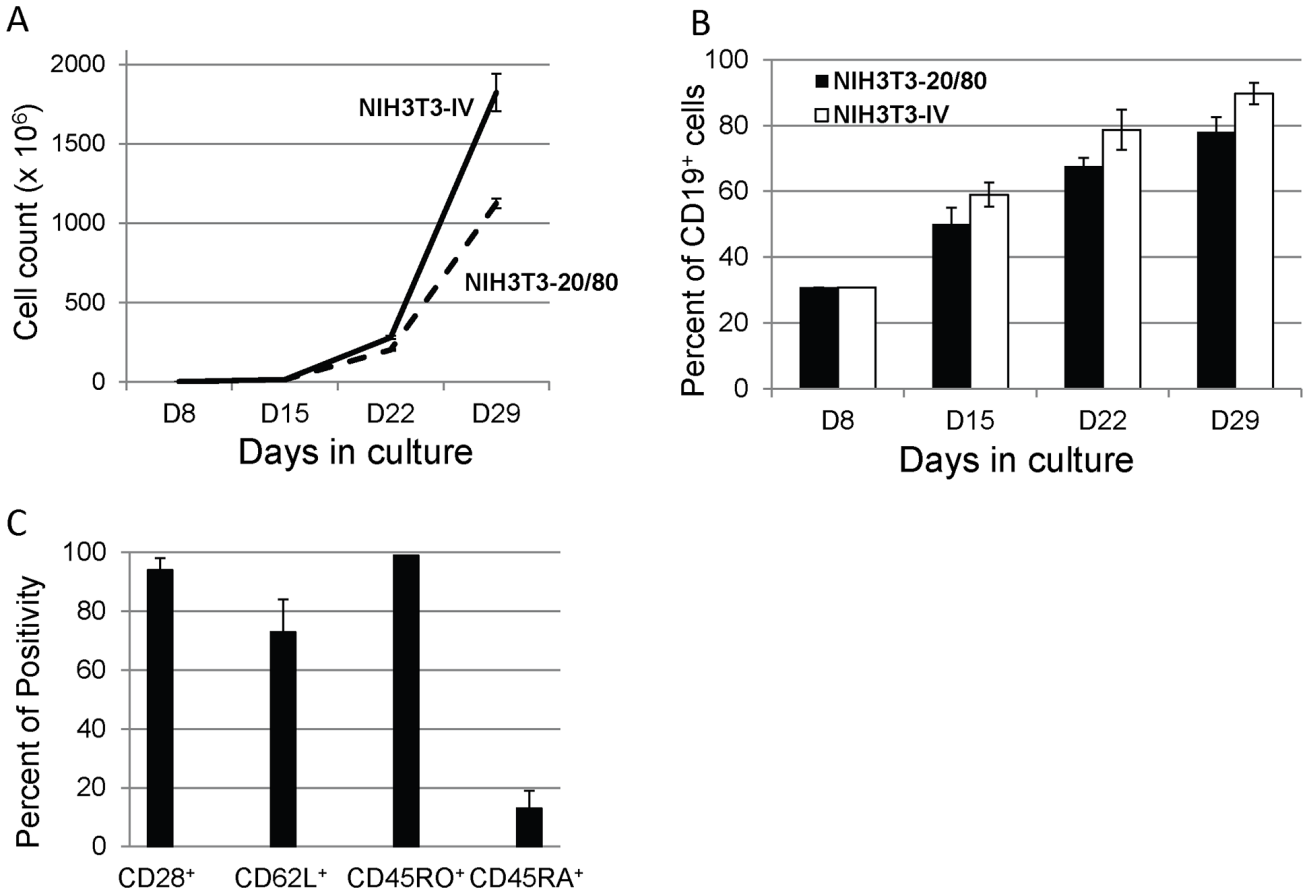
Expansion of transduced T cells using the NIH3T3-based AAPCs. (**A**) Rapid expansion of transduced T cells. Eight days after activation with CD3/28 beads, transduced T cells were cultured in plates coated with irradiated NIH3T3-20/80 (broken line) cells or NIH3T3-IV cells (solid line) in the presence of IL-2 (20 U/ml) and IL-15 (10 ng/ml on day 1 and 1 ng/ml subsequently). Cell counts were enumerated every 7 days by trypan blue exclusion. Absolute numbers of cells at various time points are depicted as mean ± SD of triplicate wells. Results are representative of three independent experiments. (**B**) Preferential enrichment of transduced T cells when co-cultured with 3T3-derived AAPCs. Eight days after activation with CD3/28 beads, transduced T cells were cultured in plates coated with irradiated NIH3T3-20/80 cells or NIH3T3-IV cells. The percentage of CD19^+^ cells was determined every 7 days by flow cytometirc analysis using a PE-conjugated mouse anti-human CD19 antibody. Results are shown as the mean percentage of CD19^+^ cells ± SD of triplicate wells at various time points and are representative of three independent experiments. (**C**) Central Memory Phenotype of expanded T cells (CD45RO^+^CD28^+^CD62L^+^). Flow cytometric analysis of T cells expanded after 2 re-stimulation cycles using NIH3T3 AAPCs was performed to determine the surface immunophenotype. The percent positivity (mean ± SD of triplicate cultures) for CD28, CD62L, CD45RO, and CD45RA was determined by corresponding antibody staining. Cells were gated on CD3^+^CD19^+^ T cells. Similar results were obtained with 4 independent experiments using cells cultured after either 2 or 3 re-stimulation cycles. Results were concordant in 5 experiments.

We constructed a series of AAPCs consisting of NIH3T3 cells transduced to express human CD20 and CD80 (NIH3T3-20/80), or human CD20, CD80, LFA-3, and ICAM-1 (NIH3T3-IV). The abilities of these AAPCs to expand and enrich i-αCD20-Δ19 T cells were tested and compared. Both NIH3T3-20/CD80 and NIH3T3-IV expanded and enriched iC9-CD20CAR-Δ19 CAR T cells efficiently. After three 7 day re-stimulation cycles, the total cell yield increased by 1124±30 fold for cultures stimulated by NIH3T3-20/80 with the percentage of CD19^+^ T cells increased by 49%±5% from initial values prior to expansion (**Figure 3A and 3B**). Addition of LFA-3 and ICAM-1 to the CD20/CD80 AAPCs (NIH3T3-IV) further improved the preferential expansion and enrichment of iC9-CD20CAR-Δ19 T cells with an 1823±113 fold increase in total cell yield, and a 59%±3% increase in the percentage of transduced T cells (**Figure 3A and 3B**).

We analyzed the phenotype of iC9-CD20CAR-Δ19 T cells at the end of each re-stimulation cycle for 3 cycles. Representative results from 5 experiments (**Figure 3C** **)** demonstrate that 95% of the expanded T cells were CD28^+^, 72% CD62L^+^, 100% CD45RO^+^ and 15% also CD45RA^+^, consistent with a central memory-like phenotype.

### CD20-specific cytotoxicity and cytokine production by iC9-CD20CAR-Δ19 T cells

**Figure 4:**
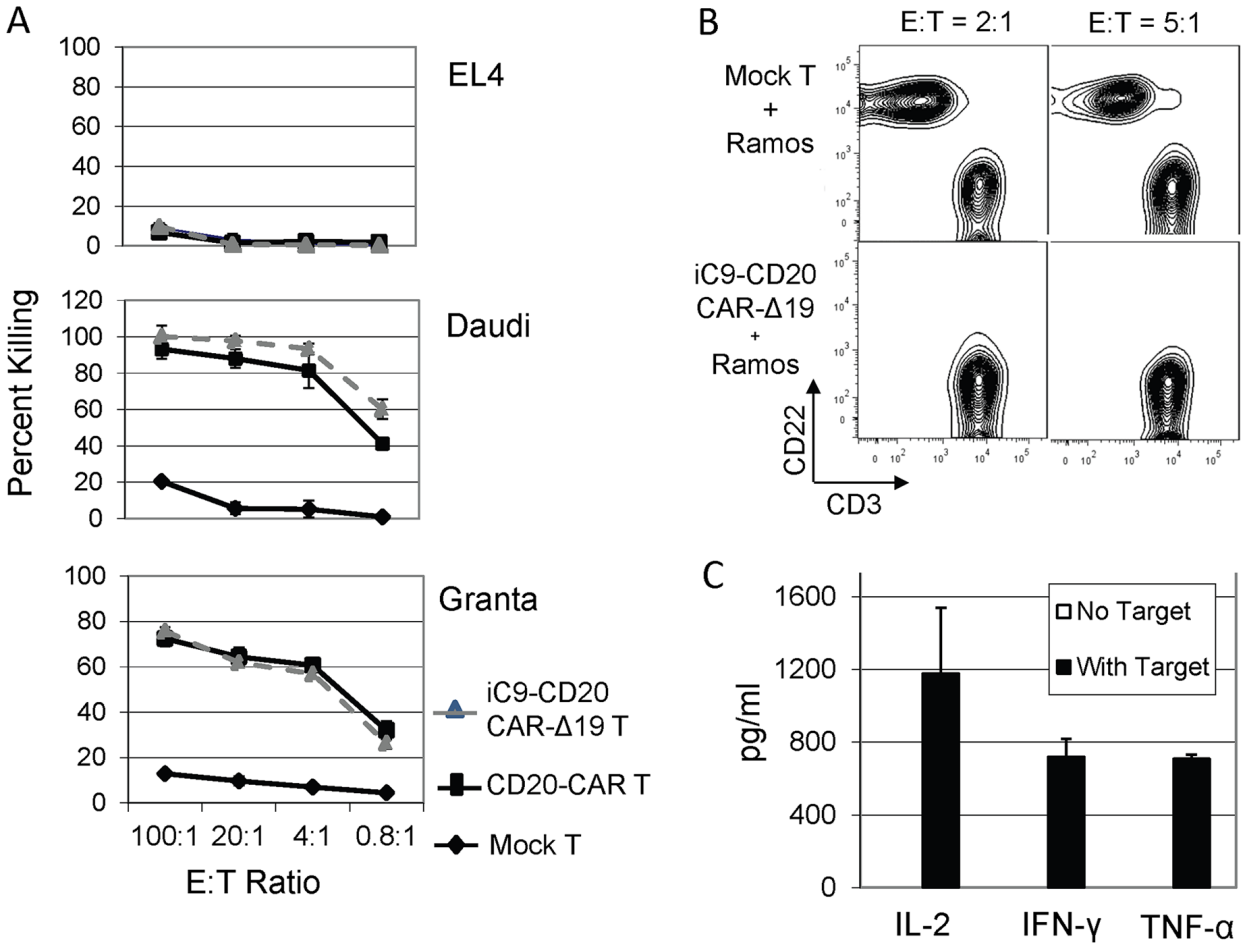
CD20-specific effector activity of transduced T cells in vitro. (**A**) Transduced T cells exhibit CD20-specific cytolytic activity in vitro. iC9-CD20CAR-Δ19 T cells transduced with CD20-CAR (CD20-CAR T), or non-transduced (mock) T cells were co-cultured with EL4, Daudi, and Granta in various effector to target (E:T) ratios. Mean percent killing (± SD of triplicate culture) was determined by 4-hour standard ^51^C release assay. (**B**) Anti-tumor effect of the transduced T cells. Mock or iC9-CD20CAR-Δ19 transduced T cells were co-cultured with CD20^+^ Ramos cells in various Effector to Target ratios (E:T) for 48 hours. Cells were then stained with antibodies recognizing CD22 and CD3. Flow cytometric analysis was used to determine the presence of Ramos cells (CD22^+^CD3^−^) and T cells (CD22^−^CD3^+^). Similar results were obtained with three independent experiments. (**C**) Cytokine production. Expanded T cells secreted IL2, IFN-γ and TNF-α after co-culture with CD20-expressing Ramos target cells for 48 hours (mean ± SD of triplicate samples). There were negligible levels of cytokine production when the expanded T cells were cultured alone with no target cells. Results are representative of three independent experiments.

We evaluated the CD20-specific cytotoxic activity of the expanded iC9-CD20CAR-Δ19 T cells using ^51^Cr-release assays and observed robust antigen-specific lysis of CD20^+^ target cells including EL4-CD20 (an EL4 line transfected with CD20, data not shown), Daudi (a human EBV lymphoma line), Raji (a human EBV^+^ lymphoma line, data not shown), Granta (a mantle cell lymphoma line) and Jeko-1 (a mantle cell lymphoma line, data not shown). All CD20-expressing target cell lines were efficiently killed even at an E:T ratio as low as 0.8 to 1 (**Figure 4A**). We also confirmed potent killing of CD20^+^ tumor cells by iC9-CD20CAR-Δ19 T cells using a complementary flow cytometric cytotoxicity assay. Specifically, T cells were co-cultured with CD20^+^ Ramos tumor cells at E:T ratios of 2∶1 and 5∶1. Residual tumor cells were distinguished from T cells in the assays using antibodies against CD22 and CD3, respectively, as Ramos cells are CD22^+^. After 48 hours of co-culture, growth of CD22^+^CD3^−^ tumor cells were observed when mock transduced T cells were used as effector cells (**Figure 4B**). In contrast, no CD22^+^CD3^−^ tumor cells were detected using iC9-CD20CAR-Δ19 T cells as effector cells (**Figure 4B**), suggesting complete CD20-specific elimination of tumor cells was achieved.

We also measured cytokine production by Elisa assay obtained 48 hours after co-culture of T cells and CD20^+^ Ramos tumor. As shown in **Figure 4C**, CAR-expressing T cells produced abundant quantities of IL-2, INF-γ, and TNF-α, demonstrating activation of both cytolytic and cytokine effector mechanisms after tumor recognition. CAR-expressing T cells alone, non-transduced T cells alone or Ramos tumor cells alone did not produce significant levels of these cytokines (Figure 4C and data not shown).

### Evaluation of anti-CD20 tumor activity in vivo

**Figure 5:**
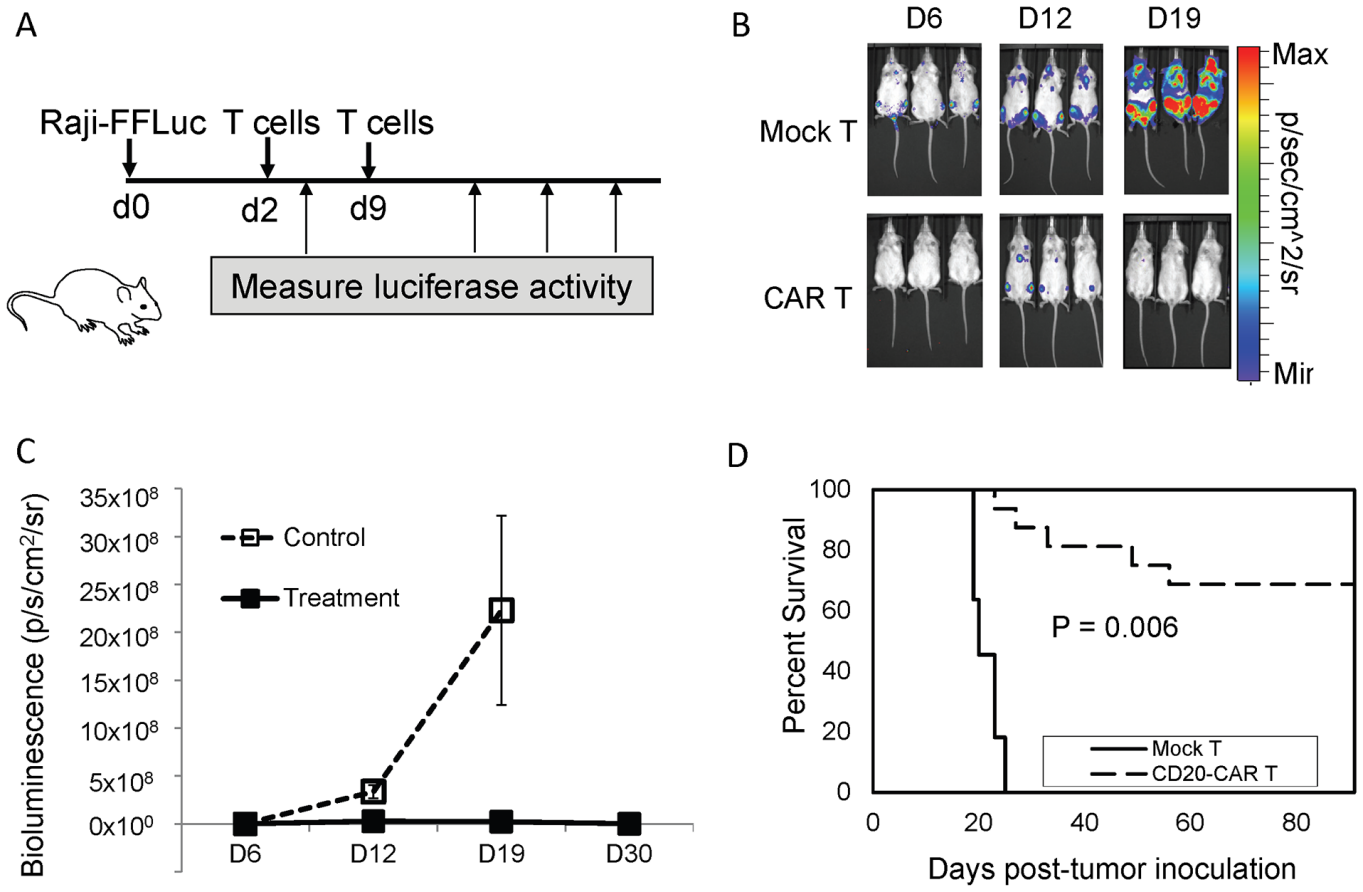
Anti-tumor activity of transduced T cells in vivo. **(A)** Schema of in vivo study demonstrating the anti-CD20 anti-tumor activity of transduced T cells in a disseminated human B cell malignancy xenogeneic NOD/SICD model. Raji-FFLuc tumor cells were injected i.v. into NOD/SCID mice on day 0. Two doses (5×10^6^ million cells per dose) of either iC9-CD20CAR- Δ19 transduced T cells or non-transduced T cells were injected i.v. on day 2 and day 9 respectively. Whole mouse luciferase activity was measured at various time points. (**B**) Eradication of CD20^+^ Raji tumors in NOD-SCID mice by CD20-CAR T cells but not non-transduced T cells (Mock T) as measured by in vivo bioluminescent imaging. (**C**) Summary of the bioluminescence signal as a measurement of tumor growth by days after tumor cell infusion. Data represent mean + SD. (**D**) Kaplan-Meier survival curves of mice receiving iC9-CD20CAR-Δ19 T treatment (CD20-CAR T) or non-transduced mock T cell treatment (NT).

In view of the potent anti-CD20 specific cytolytic activity of iC9-CD20CAR-Δ19 T cells against multiple CD20^+^ tumor lines, we next evaluated their therapeutic efficacy in vivo. We generated a CD20^+^ Raji tumor cell line with firefly luciferase expression (Raji-FFluc), which provided a non-invasive means to estimate tumor burden over time in a live mouse model (**Figure 5A**). Intravenous injection of this Raji-FFluc cell line into immunodeficient NOD/SCID mice leads to development of progressive hind-limb paralysis requiring euthanasia within 18 to 23 days. Treatment with mock-transduced T cells (5×10^6^ per injection) did not change the disease course with all control mice dying by day 23 (**Figure 5B, and 5C**). Bioluminescence was detectable on day 6 in mice in the control group (1.2×10^6^±2.5×10^5^ p/s/cm^2^/sr), rising to 3.4×10^8^±7.0×10^7^ p/s/cm^2^/sr by day 12. Extensive bioluminescence was detected on day 19 (2.2×10^9^±9.8×10^8^ p/s/cm^2^/sr in all mice in the control group (**Figure 5B, and 5C**), suggesting tumor progression was the primary cause of death. In the treatment group, no bioluminescence was detectable on day 6. Bioluminescence rose slightly to 2.8×10^7^±2.3×10^7^ p/s/cm^2^/sr on day 12 and 2.9×10^7^±3.2×10^7^ on day 19, but then decreased to 1.1×10^6^±1.2×10^6^ p/s/cm^2^/sr on day 30 (**Figure 5B, and 5C**). Eleven of 16 mice in the treatment group were alive and did not have detectable luciferase activity by the end of the 90 day observation period (**Figures 5D**). The durable complete remission rate in the treatment group was 68% compared to 0% in the control group (**Figure 5D, ***P*  =  0.006). We observed T cell persistence in peripheral blood by flow cytometry in the mice while luciferase activity was detectable, but T cells did not persist beyond the point that luciferase activity became undetectable, suggesting dependence on antigen for continued survival (data not shown).

### Elimination of iC9-CD20CAR-Δ19 T cells upon activation of the iC9 suicide gene

**Figure 6:**
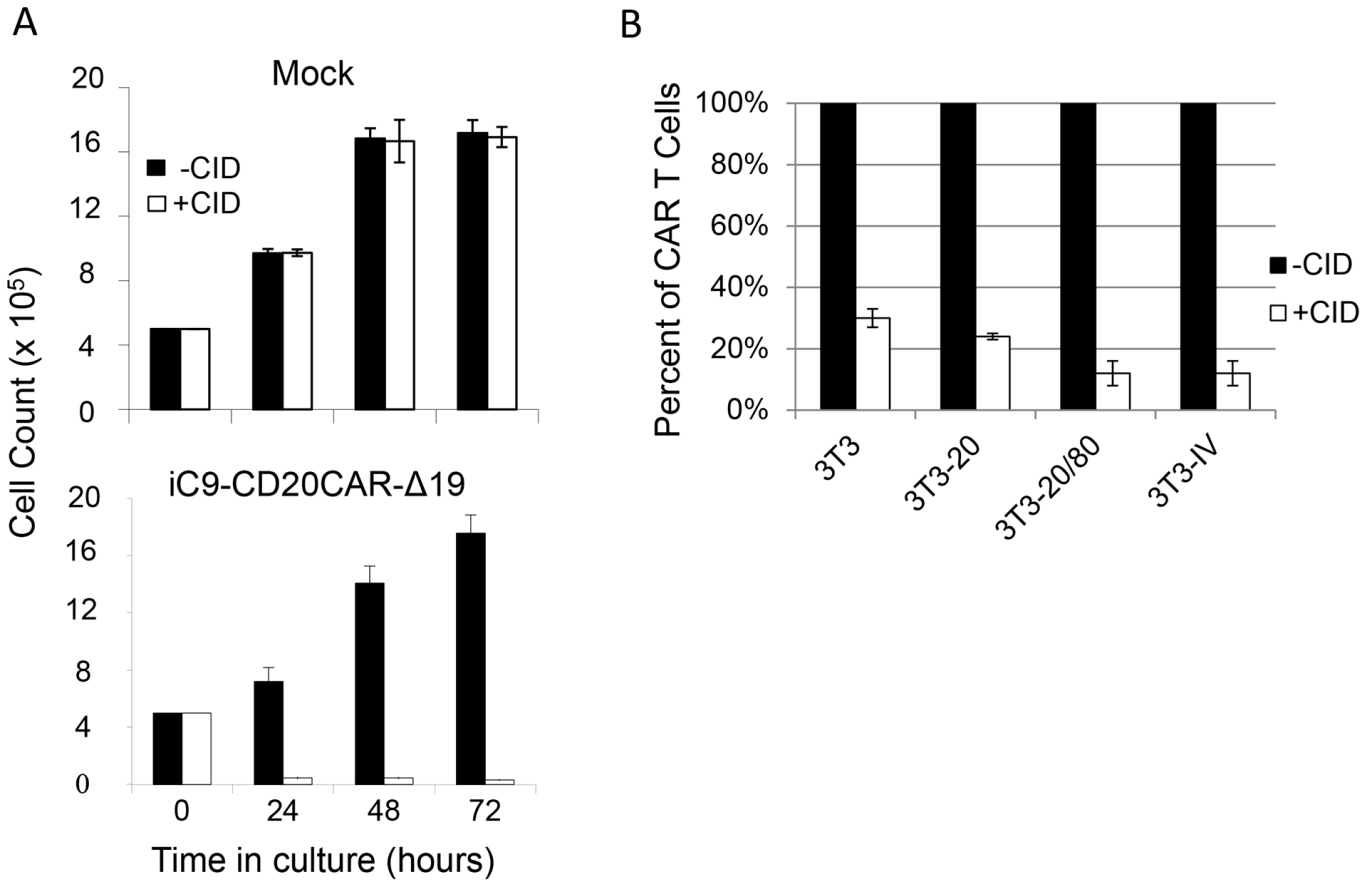
Transduced T cells are eradicated upon activation of the iC9 suicide gene. (**A**) Effect of CID on transduced T cell viability in vitro. Transduced or non-transduced (Mock) T cells were cultured in media alone (-CID) or media containing 20 nM AP1903, a clinical prodrug of CID (+CID) for 0 to 72 hours. Number of live cells was determined by trypan blue exclusion. Results are shown as mean ± SD from triplicate cultures for each condition. (**B**) Efficiency of iC9-mediated deletion is dependent on the activation status of the transduced T cells. Transduced T cells at the end of the first re-stimulation cycle using NIH3T3-20/80 were re-stimulated with either NIH3T3 cells, NIH3T3-20, NIH3T3-20/80, or NIH3T3-IV in the presence of low dose IL-2 (50 U/ml) for 60 hours. Either media (-CID) or AP1903 (20 nM, +CID) were added at 24 hours. Relative percentage of live transduced T cells was determined by flow cytometric analysis using a PE-conjugated mouse anti-human CD19 antibody. Results are shown as mean ± SD from triplicate samples for each condition.

We evaluated the efficacy of activation of the iC9 suicide gene by treating iC9-CD20CAR-Δ19 T cells with various concentrations of a chemical inducer of dimerization (CID), AP1903. As shown in **Figure 6A**, 90% of the treated T cells died within 24 hours of the initial 2 hours exposure to 20 nM AP1903 and 98% were dead after 72 hours. The CID treatment had no impact on the growth of non-transduced T cells. AP1903 was non-toxic to mock transduced T cells even at concentrations as high as 200 nM (data not shown). This observation is concordant with independent reports suggesting that the CID has no other biologic effects [32]–[34].

We next determined whether the ability of iC9-mediated T cell deletion depended on the activation status of the T cells. T cells from the end of the first re-stimulation cycle were exposed to NIH3T3 cells, NIH3T3-20, NIH3T3-20/80, or NIH3T3-IV in the presence of 50 U/ml IL-2 for 60 hours. Either culture media or AP1903 was added to the culture for the last 48 hours. Percentages of live transduced T cells were determined by flow cytometry (**Figure 6B**). There were 9%±4%, and 9%±3% of transduced T cells detected when the T cells were re-activated by either NIH3T3-20/80 or NIH3T3-IV, whereas there were 18%±2%, and 22%±1% when T cells were re-activated by NIH3T3-20 and NIH3T3 cells respectively. Hence, the efficiency of iC9-mediated T cell deletion correlates with the activation status of T cells. The more activated the T cells were, the more susceptible they were to the iC9 mediated deletion.

**Figure 7:**
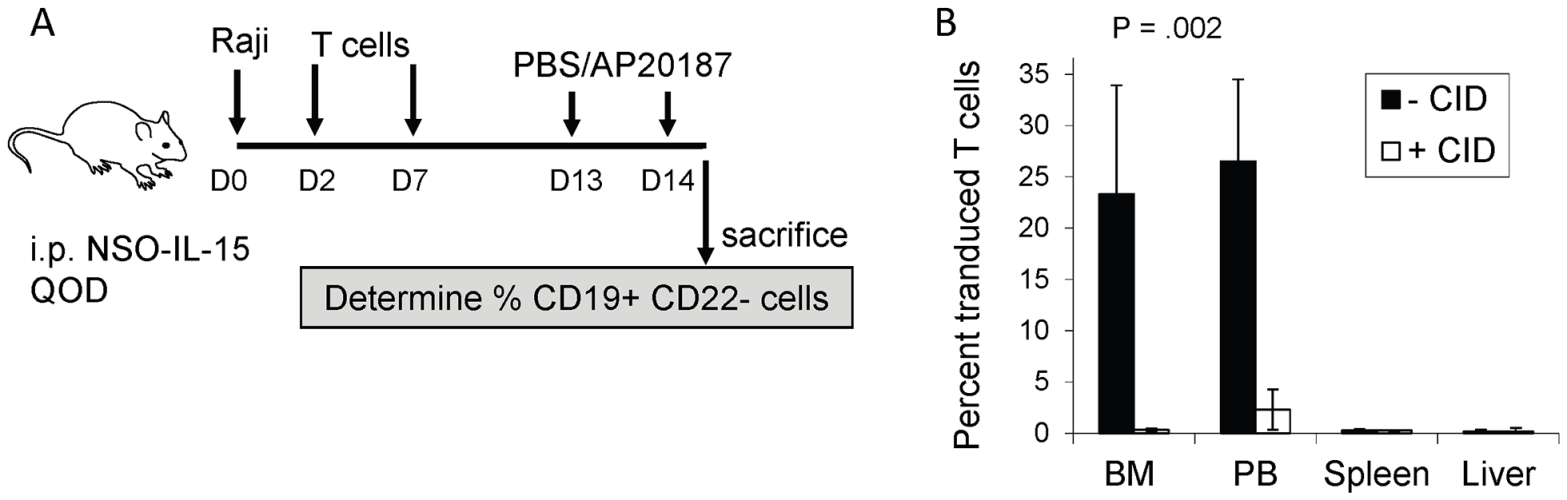
Effective removal of transduced T cells in vivo. (**A**) Study schema of *in vivo* iC9 mediated T Cell Deletion. NOD/SCID mice engrafted i.v. with 5×10^5^ Raji cells, were given two doses (1×10^7^ each dose) of iC9-CD20CAR-Δ19 T cells on Day 2 and Day7. Irradiated NSO-IL-15 cells (1.5×10^7^) were also administered i.p. every other day (QOD) to provide a source of human IL-15 in the mouse host. Two doses of PBS or AP20187 were given i.p. on days 13 and 14. Flow cytometric analysis was used to determine the percentage of transferred T cells (CD19^+^CD22^−^) in bone marrow, peripheral blood, spleen and liver. (**B**) Activation of the iC9 gene effectively removed transferred iC9-CD20CAR-Δ19 T cells in vivo. Results from the control group are displayed in closed columns (-CID). Results from the AP20187 treatment group are displayed in the open columns (+CID).

We also assessed the ability of the iC9 suicide gene to eliminate iC9-CD20CAR-Δ19 T cells in vivo *(* **Figure 7A**). To improve persistence of transferred T cells, we employed NSG mice to conduct this study. Unlike NOD/SCID mice, NSG mice do not contain residual NK cell activity and hence the transferred T cells are not eliminated by alloreaction. NSG mice were engrafted with Raji tumor cells intravenously on day 0, followed by administration of 5×10^6^ iC9-CD20CAR-Δ19 T cells intravenously on days 2 and 7. In addition, mice also received injections of irradiated NSO-IL15 cells i.p. every other day as a source of hIL-15, which improves engraftment and expansion of human memory T cells in this model [35], [36]. Two doses of either AP20187 or PBS were administered i.p. to the treatment and control groups, respectively, on days 13 and 14. AP20187 is a research grade CID with similar functional and chemical equivalence to AP1903. Twelve hours after the last dose of AP20187 or PBS, transferred T cells were quantified by flow cytometry (**Figure 7B**). In the control group, there were 26.5%±8.0% transferred T cells per 1×10^6^ total nucleated cells in blood and 23.3%±10.6% in bone marrow. In the AP20187 treated group, there were 2.5%±2.0% transferred T cells per 10^6^ total nucleated cells detected in blood, representing a 90% reduction compared to the controls. There were 0.3%±0.1% transferred T cells in bone marrow of the AP20187 treated group, representing a 98% reduction compared to controls. These results indicate that CID treatment induced effective and significant elimination of the transferred cells by activation of the iC9 gene.

## Discussion

We have presented data demonstrating the robust anti-tumor activity of T cells genetically modified to express a CD20-CAR that contains CD28 and 41BB co-stimulatory domains, and a CD3ζ endodomain. These T cells effectively lysed a variety of CD20^+^ tumor cells at low E:T ratios and secreted abundant IFNγ, TNF-α, and IL-2 upon tumor recognition, highlighting their CD20 specificity and potent effector function. More importantly, we have demonstrated for the first time to our knowledge the results of combining a third generation CAR, which has the potential for enhanced tocixity, with a conditional suicide switch utilizing the iC9 suicide gene. Our data showed deletion of 90-98% of CAR-expressing T cells upon drug induced activation of the iC9 suicide switch both in vitro and in vivo.

Our previous studies have shown that incorporation of both CD28 and 4-1BB co-stimulatory domains into the CD20-CAR T cells provides better activation, proliferation, and cytotoxicity than using CD20-CARs containing no, or single CD28 or 4-1BB domains [15]. Zhong et.al. have made similar observations in a recent study [17]. They found CAR T cells with combined CD28 and 4-1BB co-stimulatory domains and specificity for prostate-specific membrane antigen (PSMA) were superior with regards to cytokine production, in vivo survival and antigen-specific tumor elimination [17]. Superior human T cell survival and excellent antitumor activity in NSG mice was also reported by the June group [37] using CD19-CAR T cells containing 4-1BB costimulation, identifying a potential important role for incorporating 4-1BB into the CAR Although we did not perform a head-to-head comparison of the functionality between our CD28/4-1BB based CD20-CAR T cells and CD20-CAR T cells with no, or a single co-stimulatory domain in vivo, we postulate that iC9-CD20CAR-Δ19 T cells should at least outperform CAR T cells with no co-stimulatory domain.

In addition to effective cytotoxic activity, the ability of CAR T cells to persist and expand in vivo is imperative to obtain durable treatment responses. Prolonged persistence of CAR T cells has been reported in recent clinical trials using second generation CD19-CARs with either CD28 or 4-1BB co-stimulatory domains [2], [3], [5]. Our pilot clinical trial using a different third generation CD20-CAR has also documented prolonged persistence of CAR T cells [18]. Despite the differences in the CAR design, cell production methods, host conditioning, or use of cytokines, one common feature between these trials is that CD19 and CD20 are both expressed by normal B cells. Low levels of newly produced B progenitor cells might serve to provide constant antigen stimulation to maintain CAR T cell persistence in patients who achieved remission. Prolonged antigen-specific human T cell persistence was also seen in mouse xenograft tumor models, where the cells were detected in mice that still bore tumors to a variable extent [16], [37]. Therefore their persistence was tumor-dependent. We did not detect long-term CAR T cell persistence in mice achieving remission, although there were 26.8% and 23.3% CAR T cells per million nucleated cells in the blood and bone marrow, respectively (Figure 7B) 14 days after tumor engraftment at a time when tumor cells were readily detectable (data not shown). Besides lacking continued tumor antigen stimulation, other factors might also contribute to the disappearance of the CAR T cells after achievement of complete remission. For example, exogenous human cytokines such as IL-15 and IL7 have been shown to promote CAR T cell survival and persistence in the mouse environment [24]. In addition, central memory T cells have been found to have an increased ability to engraft and persist compared to other T cell subtypes in the presence of human IL-15 in mice. Most of iC9-CD20CAR-Δ19 CAR T cells had a central memory-like phenotype (CD62L^+^CD28^+^CD45RO^+^) after in vitro expansion using the NIH3T3-based AAPC expansion method, but whether these cells truly function as central memory T cells is yet to be determined. It is possible they may continue to differentiate after encountering tumor in mice and subsequently lose their ability to persist in a mouse environment.

One unique feature of our CAR T cells lies in the incorporation of a conditional iC9 suicide switch. Unlike suicide genes based on the herpes simplex virus thymidine kinase (HSV/TK) gene product that are immunogenic, the iC9 gene is fully human, with the only potential immunogenic epitopes being at fusion sites with FKBP. Moreover, iC9 has safely mediated highly efficient and specific deletion of transduced cells in both preclinical and clinical studies [25], [34]. We have observed eradication of >90% of the iC9-CD20CAR-Δ19 T cells upon activation of iC9 both in vitro and in vivo in the current study. Therefore, incorporation of iC9 may enable us to reverse potential adverse events associated with transferred iC9-CD20CAR-Δ19 T cells such as profound B cell aplasia, hypogammaglobulinemia, severe infusion reactions, and dysregulated T cell proliferation due to insertional mutagenesis. The CID used to trigger the suicide gene product is a biologically inert prodrug. Unlike ganciclovir, it does not have other biological effects or cause any known potential serious adverse events, and has been well tolerated in clinical studies [25], [38]. It activates the mitochondrial apoptotic pathway and hence its activity in the target cells is not cell cycle dependent. This explains its rapid onset of action. However, induction of cell death requires expression of the iC9 gene above a certain threshold level. We were not able to elicit efficient elimination of the CAR T cells when we tested lentiviral constructs in which the iC9 gene was not placed immediately at the 5’ end of the insert despite using 2A linkers to improve the expression level (data not shown). The efficiency of iC9 gene-mediated T cell deletion also correlated with the activation state of the T cells. Fewer iC9-CD20CAR-Δ19 T cells were deleted by the iC9 system when the T cells were not activated or were suboptimally activated. This observation corroborates findings by Tey et al [34], who found that iC9 gene expression and function were restored upon cell reactivation in vitro. Recently Di Stasi et al. observed that the majority of T cells that survived iC9-mediated deletion in vivo, when expanded and reactivated in vitro, were rapidly deleted upon exposure to CID [25]. Most, if not all of the reported complications of transferred CAR-T cells in clinical trials have been attributed to activated T cells. Therefore, even if the iC9 suicide system is not able to remove 100% of resting T cells, these T cells might not cause significant deleterious effects in vivo. To further increase the efficacy of the iC9 system, multiple doses of CID could be applied at various time points to achieve the desired elimination. Alternatively a CD19-drug conjugate could be tested to remove the residual iC9-CD20CAR- Δ19 transduced T cells that survived iC9-mediated deletion as these T cells maintained low level of CD19 expression on the surface.

The current study provides the preclinical basis for a planned clinical trial testing adoptive T cell immunotherapy for patients with CD20+ malignancies in a safer and more efficient manner.
